# Dose-dependent effects of neem crude extract on human dental pulp cell and murine osteoblast viability and mineralization

**DOI:** 10.1590/0103-6440202205207

**Published:** 2022-12-05

**Authors:** Somying Patntirapong, Visakha Aupaphong, Patcharin Pipatboonyarit, Kasira Kritsuttsikun, Thanyaporn Phubai

**Affiliations:** 1 Thammasat University Research Unit in Dental and Bone Substitute Biomaterials, Faculty of Dentistry, Thammasat University, Pathumthani, Thailand; 2 Faculty of Dentistry, Thammasat University, Pathumthani, Thailand

**Keywords:** neem, dental pulp cell, osteoblast, viability, mineralization

## Abstract

Neem has been used as a medicine due to its beneficial properties such as anti-microbial effects. Neem products for oral application are on the rise. Before recommendation for therapeutic use in human, its effects on cellular activities need to be examined. Therefore, the aim of this study was to test the effects of the ethanolic neem crude extract on dental pulp cells and osteoblasts in terms of cell viability, mineralization, and gene expressions. The ethanolic neem extract derived from dry neem leaves was subjected to chemical identification using GC-MS. Human dental pulp stem cells (hDPSCs) and pre-osteoblasts (MC3T3) were treated with various concentrations of the neem crude extract. Cell viability, mineralization, and gene expressions were investigated by MTT assay, real-time PCR, and alizarin red S assay, respectively. Statistical analysis was performed by one-way ANOVA followed by Dunnett test. GC-MS detected several substance groups such as sesquiterpene. Low to moderate doses of the neem crude extract (4 - 16 µg/ml) did not affect hDPSC and MC3T3 viability, while 62.5 µg/ml of the neem extract decreased MC3T3 viability. High doses of the neem crude extract (250 - 1,000 µg/ml) significantly reduced viability of both cells. The neem crude extract at 1,000 µg/ml also decreased viability of differentiated hDPSC and MC3T3 and their mineralization. Furthermore, 4 µg/ml of neem inhibited viability of differentiated hDPSC. There is no statistical difference in gene expressions related to cell differentiation. In conclusion, the neem crude extract affected cell viability and mineralization. Cell viability altered differently depending on the doses, cell types, and cell stages. The neem crude extract did not affect cell differentiation. Screening of its effect in various aspects should be examined before the application for human use.

## Resumo

O Neem tem sido utilizado como medicamento devido às suas propriedades benéficas, tais como os efeitos antimicrobianos. Os produtos Neem para aplicação oral estão a aumentar. Antes da recomendação para uso terapêutico no ser humano, os seus efeitos nas atividades celulares precisam ser examinados. Por conseguinte, o objectivo deste estudo era testar os efeitos do extracto bruto de neem etanólico nas células de polpa dentária e osteoblastos em termos de viabilidade celular, mineralização e expressões genéticas. O extracto de neem etanólico derivado de folhas secas de neem foi sujeito a identificação química utilizando GC-MS. As células estaminais de polpa dentária humana (hDPSCs) e os pré-osteoblastos (MC3T3) foram tratados com várias concentrações do extrato bruto de neem. A viabilidade celular, mineralização, e expressões genéticas foram investigadas pelo ensaio MTT, PCR em tempo real, e o ensaio S vermelho de alizarina, respectivamente. A análise estatística foi realizada por ANOVA unidirecional seguida pelo teste Dunnett. O GC-MS detectou vários grupos de substâncias como o esquisterpeno. Doses baixas a moderadas do extracto bruto de neem (4 - 16 µg/ml) não afetaram a viabilidade do hDPSC e MC3T3, enquanto 62,5 µg/ml do extracto de neem diminuiu a viabilidade do MC3T3. Doses elevadas do extrato bruto de neem (250 - 1.000 µg/ml) reduziram significativamente a viabilidade de ambas as células. O extrato bruto de neem a 1.000 µg/ml também diminuiu a viabilidade de hDPSC e MC3T3 diferenciados e a sua mineralização. Além disso, 4 µg/ml de neem inibiu a viabilidade do hDPSC diferenciado. Não há diferença estatística nas expressões genéticas relacionadas com a diferenciação celular. Em conclusão, o extrato bruto do neem afetou a viabilidade celular e a mineralização. A viabilidade celular alterou-se diferentemente dependendo das doses, tipos de células, e fases celulares. O extrato bruto do neem não afetou a diferenciação celular. O rastreio do seu efeito em vários aspectos deve ser examinado antes da aplicação para uso humano.

## Introduction

Neem or *Azadirachta indica* is a tree that belongs to the family Meliaceae. It is a native tree grown in tropical and semi-tropical regions such as India, Pakistan, Bangladesh, Nepal and Thailand. Neem has drawn attention in traditional and modern medicinal fields since this tree is rich in bioactive compounds ^1^ ^-^ ^3^. Several parts of neem including leaves, seeds, bark, twig, root and flower can be applied for biological and pharmacological purposes ^3^ ^-^ ^7^. 

Neem extract has therapeutic potential in human subjects. Leaf extract from neem accelerated healing ability in rat skin lesion via inflammatory response and neovascularization ^3^. Neem exhibited an anti-bacterial activity against the bacteria *Vibrio vulnificus* that causes sepsis, severe cellulitis, fever, vomiting, and necrotizing fasciitis ^8^. Furthermore, many studies have attempted to develop neem products for oral use since neem possesses broad range of anti-microbial properties (9-12). Neem extracts from leaf and twig inhibited growth of two periodontal pathogens namely *Prevotella intermedia* and *Fusobacterium nucleatum* ^5^. Neem leaf extract showed inhibitory effect against dental caries and endodontic pathogens such as *Streptococcus mutans*, *Staphylococcus aureus* and *Enterococcus faecalis* ^6^. When applied the substance as an irrigating solution, it showed anti-microbial activity against *E. faecalis*. Its efficacy was comparatively similar to that of sodium hypochlorite irrigating solution, which is the gold standard of root canal irrigant ^11^ ^,^ ^12^.

In the dental field, the oral use of the neem extracts can potentially be employed in endodontic irrigants, pulp capping, and mouth rinse ^10^ ^-^ ^12^. Neem-based mouth rinse was efficient in reducing gingivitis ^9^. However, with such applications, it was found that neem-based mouth rinse could cause adverse events such as burning sensation and epithelium desquamation in some subjects ^9^. This drives the need to screen for its cytotoxicity and effects on cellular activities before it can be recommended for clinical use in human. In order to use neem as the potential pulp capping and endodontic irrigant, cells that could be exposed to these treatments are dental pulp cells and osteoblasts, respectively. To our knowledge, the effects of the neem crude extracts on dental pulp cells and osteoblasts have not been studied. Therefore, we tested whether an ethanolic neem extract affected dental pulp cells and osteoblasts in terms of cell viability, mineralization, and gene expressions of odontoblast and osteoblast markers. The null hypothesis of this study was that cell viability, mineralization, and gene expressions of odontoblast and osteoblast markers were not different after ethanolic neem extract treatment.

## Materials and methods

### Neem extraction

Mature fresh neem leaves identified as *Azadirachta indica A. Juss. var. Siamensis Valeton.* were collected from a farm in Thailand. Fresh neem leaves were washed and dried by 2 means: under the shade for 4 h and under the oven at 50°C for 1 h. Dried leaves were crushed into crude powder and subjected for extraction with absolute ethanol. The mixture was incubated in shaker incubator for 24 h, boiled in hot water bath for 30 min and incubated in shaker incubator for 24 h. The resulting solvent was filtered through Whatman No.1 filter paper and evaporated by rotary evaporator under reduced pressure at 40°C to obtain the crude extract. Then, the crude extract was subjected to phytochemical identification by gas chromatography-mass spectrometry (GC-MS) as previously described ^13^. Identification of compounds was achieved by comparing them with the MS known library.

### Cell culture and treatments

The Research Ethics Committee of Thammasat University (Project No. 105/2565) approved this study. Poietics™ human dental pulp stem cells (hDPSCs) and pre-osteoblasts (MC3T3) were obtained from Lonza and ATCC, respectively. hDPSCs are multipotent stem cells harvested from pulp tissue of adult wisdom teeth or third molars, while MC3T3 were isolated from the calvaria of mouse. hDPSCs were maintained in Dulbecco's Modified Eagle Medium, while MC3T3 were maintained in alpha-minimum essential at 37°C and 5% CO_2_ humidified atmosphere. Both media were supplemented with 10% fetal bovine serum and 1% penicillin/streptomycin (standard culture media; all from Gibco, Invitrogen). hDPSCs (passage 3-5) and MC3T3 (passage 26-28) were used for the experiments. 

Cells were verified for the sub-confluency and their morphology with a microscope before starting the test. When the cells reached 80% confluency on T-75 flasks, the cells were trypsinized and seeded on 96-well plates. For the cytotoxicity test, a sub-confluent single cell layer was prepared. hDPSCs and MC3T3 were plated at the density of 1.5 x 10^4^ cells/cm^2^ and 8 x 10^3^ cells/cm^2^, respectively. The cells were incubated in the standard culture media at 37°C and 5% CO_2_ humidified atmosphere for 24 h. Afterwards, the culture media were replaced with the neem crude extract in the standard culture media at various concentrations (4, 16, 62.5, 250, and 1,000 µg/ml) and incubated for 3 days. The neem crude extracts at concentrations of 4 - 16 and 250 - 1,000 µg/ml were considered low and high concentrations, respectively, in this experiment.

For odontogenic differentiation of hDPSCs, the culture media were changed to odontogenic media (ODM), which consisted of the standard culture media with 50 µg/ml ascorbic acid (BDH), 10 mM β-glycerophosphate (Sigma), and 100 nM dexamethasone. For osteogenic differentiation, MC3T3 cells were induced with osteogenic media (OM), which were the standard culture media containing 50 μg/ml ascorbic acid and 2 mM β-glycerophosphate. The cells were treated with the neem crude extract (4 and 1,000 µg/ml) for 14 and 21 days for real-time PCR and alizarin red S assay, respectively. The cells without neem addition were served as a control. 

### Cell cytotoxicity assay

The determination of cytotoxicity was done according to ISO 10993-5:2009(E). Before cytotoxicity testing, cell morphological analysis was examined under a light microscope (Nikon Eclipse TS100, magnification 100x) and recorded using a Nikon Digital sight DS-L2. Cell cytotoxicity was examined using a 3-(4,5-dimethylthiazol-2-yl)-2,5-diphenyltetrazolium bromide (MTT; Sigma). After treatments, the media with 0.2% MTT solution were added and incubated at 37ºC for 4 h. The media were discarded and the reaction was stopped with dimethylsulfoxide (DMSO) and glycine buffer (all from Sigma). The end product color was measured at 620 nm absorbance (OD).

### RNA isolation and polymerase chain reaction (PCR)

The expressions of three key genes associated with cell differentiation were examined by real-time PCR. First, total RNA was isolated from the cells treated with the neem crude extract for 14 days using Total RNA Mini kit (Geneaid). The same amount of mRNA from each sample was reverse-transcribed into cDNA using PrimeScript^TM^ RT reagent Kit (TaKaRa). 

For PCR, an aliquot of each cDNA sample was amplified using KAPA SYBR^®^ FAST qPCR Master Mix (KAPABIOSYSTEMS). The cycle conditions were set up as detailed: 50 °C for 2 min initial heating, 95 °C for 1 min, 40 cycles of 95 °C for 30 s followed by 60 °C for 30 s with 72 °C elongation for 30 s each, melt curve stage 95°C for 30 s followed by 55 °C for 1 m and 95 °C for 1 m. The PCR was carried out in an Applied biosystems QuantStudio3 Realtime PCR system (Applied Biosystems; ABI). The forward/reverse primer pairs were shown in Box 1. Glyceraldehyde 3-phosphate dehydrogenase (GAPDH) was used as an internal control gene.

**Box 1:**
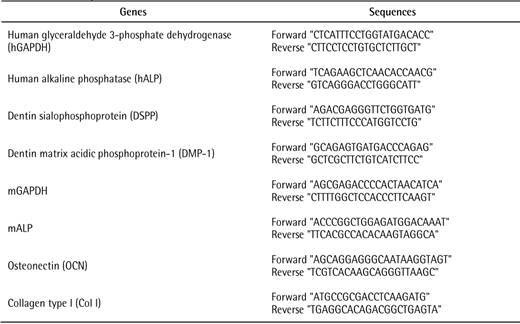
Primer sequences

### Alizarin red S (ARS) assay

After 3 weeks of culture in the continuing presence of ODM or OM and the neem crude extract, the cells were washed with PBS, fixed with ice-cold ethanol for 10 min, and washed with distilled water. Freshly prepared 1% ARS (Sigma) was added into the wells and incubated at room temperature for 10 min. The cells were then thoroughly washed to remove the excess dye with distilled water and air-dried. Mineralized nodule formation was seen as bright red-colored deposition throughout the wells. Photomicrographs were captured. For quantification, the cells positively stained with ARS were destained with 10% cetylpyridinium chloride (Sigma), and the extracted stain was measured at the 570 nm absorbance.

### Statistical analysis

All experiments were conducted in three independent experimental sets. Each set was done in duplicate. The data are expressed as means ± SD. Normality of the data were tested by Shapiro-Wilk test. Statistical analyses were performed using one-way ANOVA followed by Dunnett multiple comparison test (GraphPad Prism 5, GraphPad Software Inc., La Jolla, CA, USA). *p*<0.05(*), *p*<0.01(**) and *p*<0.001(***).

## Results

### Preliminary chemical screening by GC-MS

GC-MS allowed the detection of various substance groups including sesquiterpenoids, diterpenes, terpenoid, organoheterosilanes, trialkylamines, ethylamines, fatty acid, amino acid, carboxylic acids, hydrocarbon, benzofuran, barbiturate, glucoside and furanoid. From the ethanolic neem extract, 35 compounds were identified ^13^. Nine out of thirty-five compounds were phytol, α-capaene, α-humulene, β-caryophyllene, δ-cadinene, carvone, aromadendrene, octadeamethyl-cyclononasiloxane, and β-bourbonene. According to previous studies, these compounds possessed the anti-proliferative property ^13^ ^-^ ^21^ (see detail in Discussion). One compound, β-caryophyllene, possessed mineralization induction property ^22^. The rest have anti-bacterial, anti-fungal, anti-viral, anti-inflammatory, anti-oxidant and anti-diabetic properties. 

### Effects of the neem crude extract on cell growth

**Figure 1:**
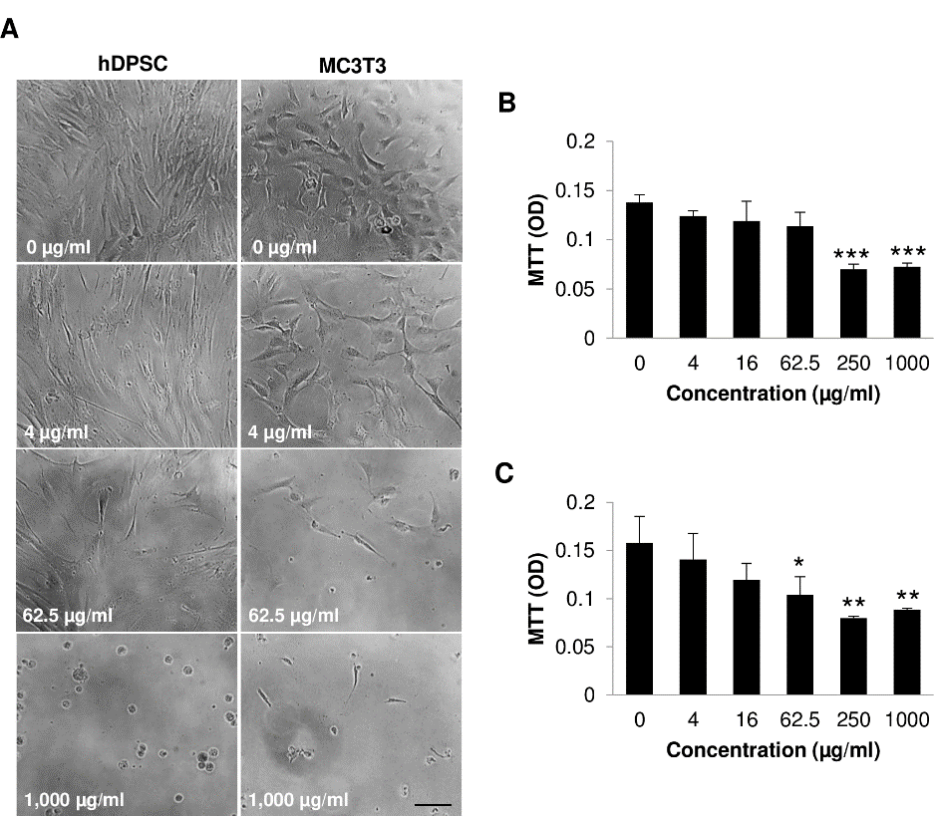
Assessment of cell viability and morphology. Cells were treated with the neem crude extract at various concentrations ranging from 4 to 1,000 µg/ml for 3 days. (A) Representative micrographs of cell morphology treated with the neem crude extract at 0, 4, 62.5, and 1,000 µM. Scale bar = 200 µm. (B) MTT assay of hDPSCs (C) MTT assay of MC3T3

hDPSCs and MC3T3 were first treated with the neem crude extract at 0, 4, 16, 62.5, 250, and 1,000 µg/ml for 3 days to screen for the applicable dose level. In general, the untreated cells spread well throughout the culture well. Untreated hDPSCs were fibroblast-like morphology with cell density at about 70% confluency, while untreated MC3T3 were polygonal appearance with cell density at 60% confluency. Cell shape and cell confluency were noticeably altered after treatment with the neem extract at concentrations higher than 16 µg/ml. At 1,000 µg/ml, more than 70% of cells shrank and appeared as round shape (cell death) (Figure 1A). The neem crude extract at 4 - 62.5 µg/ml did not affect cell viability of hDPSCs. At concentration 250 and 1,000 µg/ml, the neem crude extract significantly reduced hDPSCs viability. Cell viability was reduced to approximately 51 and 53% of the untreated control, respectively (Figure 1B). Cell viability of MC3T3 was significantly decreased when treated with the neem extract at concentration of 62.5 - 1,000 µg/ml (Figure 1C). According to the ISO 10993-5:2009(E) recommendation, 250 and 1,000 µg/ml of neem crude extract were cytotoxic (more than 30% reduction in cell viability). The neem crude extracts at concentrations of 4 and 1,000 µg/ml were selected for latter experiments because of its non-cytotoxic and cytotoxic effects, respectively. 

### Effects of the neem crude extract on bone nodule formation

**Figure 2:**
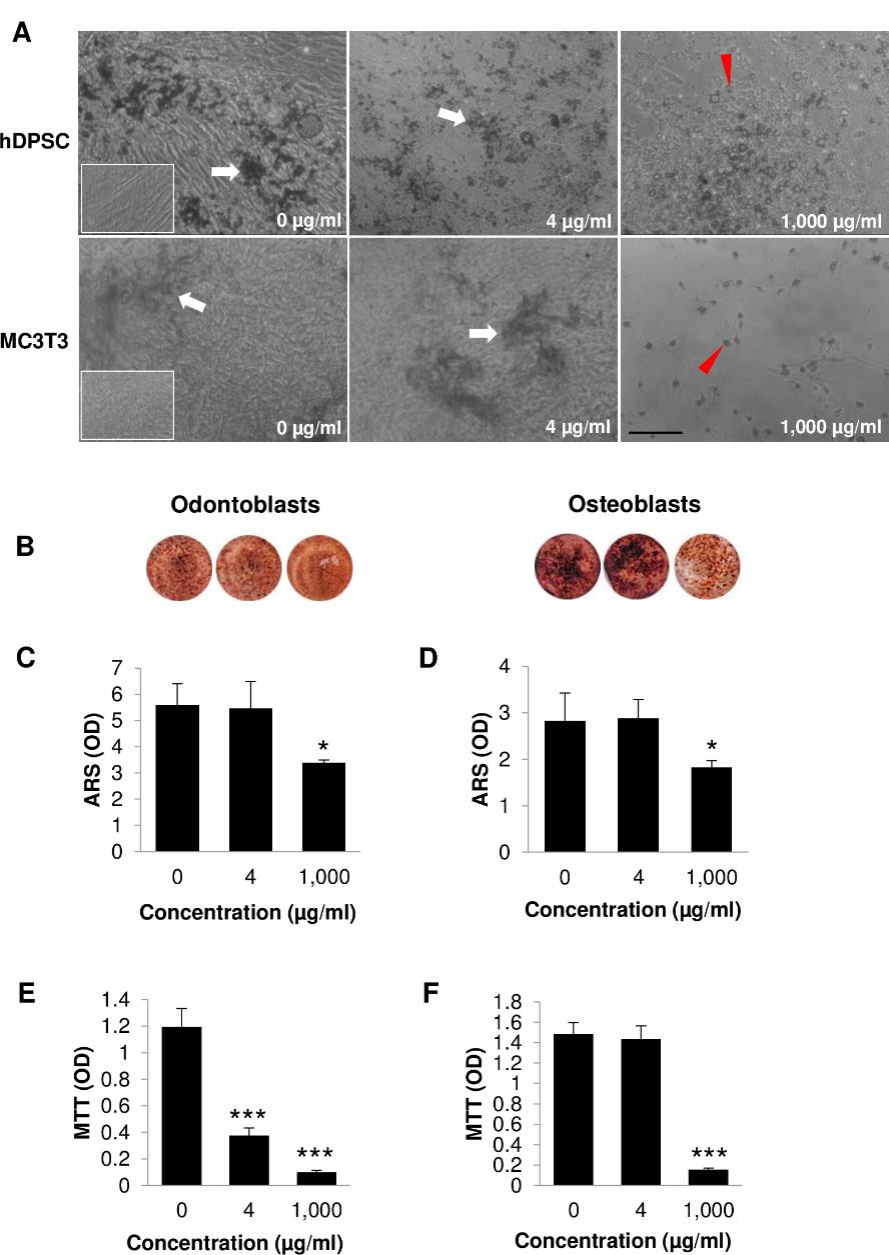
Effects of the neem crude extract on mineralization and cell viability. Cells were treated with the neem crude extract for 21 days. (A) Representative micrographs of cells treated with the neem crude extract at 0, 4, and 1,000 µM. White arrows demonstrate nodule formation. Red arrow heads showed cell death (round shape). Insets were cells cultured in the standard culture media (no nodule formation). Scale bar = 200 µm. (B) Alizarin staining (C) Total ARS quantification of odontoblasts (D) Total ARS quantification of osteoblasts (E) Cell viability of odontoblasts (F) Cell viability of osteoblasts

Both cells are capable of forming bone nodule when induced with ODM/OM media as indicated by white arrows (Figure 2A; left panel). Odontoblasts and osteoblasts covered throughout the well surface (100% confluency). The cells in standard media showed no nodule formation (Inset; Figure 2A). Dead cells were observed in samples treated with 1,000 µg/ml neem crude extract as indicated by the red arrow heads (Figure 2A; right panel). The bone nodules were positive to ARS and were monitored throughout the culture wells (Figure 2B). The OD value of mineralization treated with high concentration of neem crude extract significantly reduced compare with that of the control (Figure 2C and D). When examining cell viability at 21 days, the result showed that 4 and 1,000 µg/ml neem crude extract significantly inhibited odontoblast viability, while 1,000 µg/ml neem crude extract significantly reduced osteoblast viability compared with control (Figure 2E and F). 

### Effects of the neem crude extract on mRNA expressions

**Figure 3:**
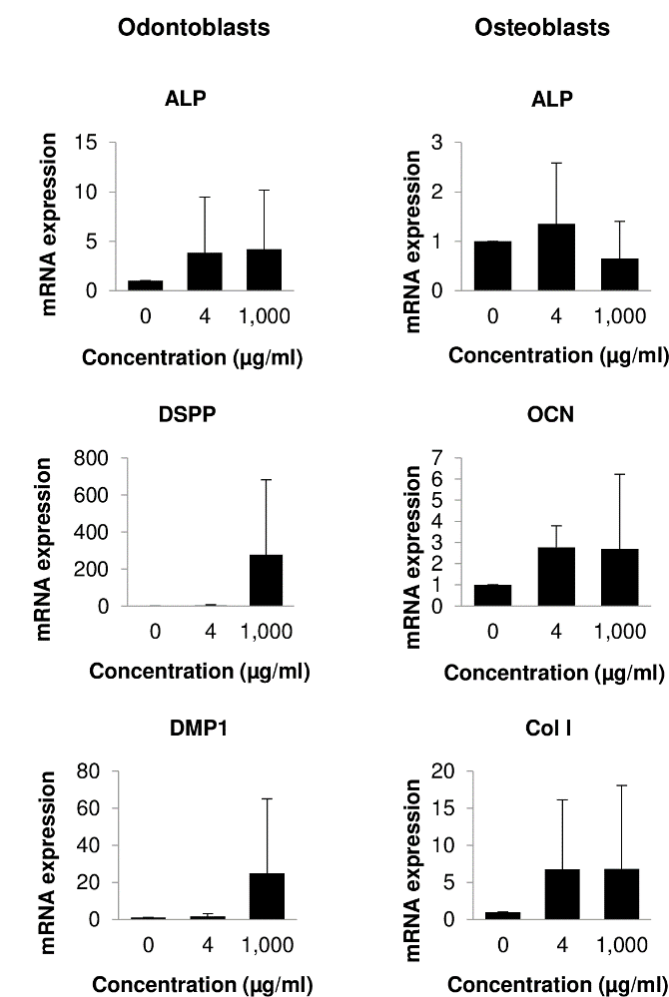
Investigation of gene expressions. Cells were treated with the neem crude extract for 14 days. mRNA expressions were examined by qPCR. The left column is odontoblast gene expressions. The right column is osteoblast gene expressions

In odontoblasts after 14 days, the results showed that 1,000 µg/ml neem extract-treated cells tended to have higher ALP, DSPP, and DMP-1 mRNA expressions than control. In osteoblasts, 4 µg/ml neem extract-treated cells trended to have higher ALP, OCN, and Col I mRNA expressions than control (Figure 3). However, there was no statically significant difference in gene expressions.

## Discussion

In this study, the neem crude extract differently affected viability of both precursors and differentiated cells of dental pulp and pre-osteoblasts. Furthermore, mineralization was reduced. Therefore, the null hypothesis of the present study was rejected. 

In undifferentiated hDPSC and MC3T3 cells, high concentration of neem inhibited cell viability more than 50% and caused cell death. However, low concentration of neem did not reduce cell viability and did not affect cell morphology. The concentration that hDPSC and MC3T3 cells were still viable at 50% was 250 µg/ml. In differentiated hDPSC and MC3T3 cells, high concentration caused pronounced cytotoxicity in both cells. Nevertheless, low concentration reduced cell viability only in differentiated hDPSCs. Neem also affects other cell types. It caused cytotoxicity to human lymphocytes at concentrations of 1.2 - 2 mg/ml after 24-hour treatment. Because neem generates oxidative stress, which subsequently damages DNA ^8^. The anti-proliferative effect of the neem leave extract was demonstrated in human breast cancer cell line (MCF-7) and human cervical carcinoma cell line (HeLa). However, it should be noted that the dose and time for induction of cell death are different. The EC_50_ is at 350 µg/ml for 3 days in MCF-7 cells, while it is at 175 µg/ml for 48 h in HeLa cells ^23^. These data implied that viability of cells was dependent on the doses, cell types, and stages of cells. 

The presence of a single compound induces inhibition of cell proliferation (14-18,21). For example, α-humulene (a sesquiterpene) prevented hepatocellular carcinoma cell proliferation and caused cell apoptosis via the inhibition of Akt signaling ^14^. β-caryophyllene (another sesquiterpene) as a single compound showed anti-proliferative effects on 2 tumor cell lines. This compound acted through caspase-3 activity ^17^. Interestingly, β-caryophyllene potentiated the cytotoxic ability of α-humulene. The addition of β-caryophyllene to α-humulene increased cell growth inhibition on the human breast adenocarcinoma cell line (MCF-7) than α-humulene treatment alone ^24^. It was found that 9 compounds related to the inhibition of cancer cell growth *in vitro* ^14^ ^-^ ^21^ or caused apoptosis in cancer in Swiss albino mice ^25^. In the neem crude extract, these compounds comprised of approximately 25.22% of all compounds combined. It was possible that these compounds worked in concert in inhibition of dental pulp and osteoblastic cell growth. 

Total mineralization was reduced when high dose of neem was added to both cell types compared to control. In contrast to this data, neem-derived triterpenoids possess osteogenic activity. Three triterpenoids (Azadirone, Azadirachtin A and Azadirachtin B) enhanced osteoblast mineralization *in vitro* at the different concentrations. Azadirachtin A stimulates osteogenic gene expressions and bone formation *in vivo* by binding selectively to a site in the estrogen receptor ^26^ ^,^ ^27^. The different in the results was possibly due to isolated compounds in previous studies versus crude extract in this study. Azadirachtin could not be detected by GC-MS method. Other methods should be tested to identify the substances and concentration from the crude extract in the future.

Although a number of cell viability (90%) at 21 days was reduced by high dose of neem, approximately 40% of mineralization was decreased. This implied that the reduction of nodule formation was in part by the inhibition of cell viability. Less viable cells lead to less total mineralization. It is worth noting that a portion of the cell population was still viable. These cells seemed to maintain the differentiation stage and the mineralization ability. β-caryophyllene was presented in the neem crude extract. A pure compound stimulated mineralization of osteoblasts isolated from bone marrow ^22^. It might be possible that this compound partly enhanced mineralization in viable cells. Since the crude neem extract contained several compounds, therefore, it was difficult to pin-point a single compound that could affect cell activities. 

The use of neem for clinical application in the dental field is beneficial. However, some drawback should be considered before clinical use. The possibility that cells in the oral cavity may expose to the neem extract increases when applying near the pulp or irrigating in the pulp cavity. The effective dose without toxicity, treatment time, and duration of application might vary among different cell types. Accordingly, this study demonstrated that neem crude extract at lower concentration was nontoxic, while the high concentration inhibited cell growth and mineralization. Thus, low concentration was an appropriate dose that might be used for an application strategy. It is necessary to assess cytotoxicity and safety issues of these extracts before applying for preclinical and clinical trials i.e., an endodontic irrigant or intracanal medicament ^6^.

This study had limitations. Firstly, the crude extract contained many types of compounds. Therefore, the result came from the combination of compounds. In the future, an active compound should be isolated individually and tested on cells. Secondly, the concentrations and frequency that cells may expose to *in vivo* are not known yet and might not be equal to the concentrations and frequency used in this study.

## Conclusion

This study reported that high concentration of neem crude extracts inhibited cell growth and reduced mineralization. The non-cytotoxic concentration did not affect cell growth and mineralization. Since neem extract is a promising traditional medicine for many therapeutic applications, the potentiating effects of the extract should be evaluated before going on for further preclinical and clinical use in human. 

## References

[1] Shah S, Venkataraghavan K, Choudhary P, Mohammad S, Trivedi K, Shah SG (2016). Evaluation of antimicrobial effect of azadirachtin plant extract (Soluneem (TM)) on commonly found root canal pathogenic microorganisms (viz. Enterococcus faecalis) in primary teeth: A microbiological study. J Indian Soc Pedod Prev Dent.

[2] Kim JG, Son KM, Park HC, Zhu T, Kwon JH, Yang HC (2013). Stimulating effects of quercetin and phenamil on differentiation of human dental pulp cells. Eur J Oral Sci.

[3] Emeka O, Emamoke O, Theodore A, Julius O (2013). The Wound Healing Effects of Aqueous Leave Extracts of Azadirachta Indica on Wistar Rats. J Nat Sci Res.

[4] Schumacher M, Cerella C, Reuter S, Dicato M, Diederich M (2011). Anti-inflammatory, pro-apoptotic, and anti-proliferative effects of a methanolic neem (Azadirachta indica) leaf extract are mediated via modulation of the nuclear factor-kappaB pathway. Genes Nutr.

[5] Parashuramaiah SC, Chanu TP (2019). Effect of Neem leaf and Neem Twig Extract on Prevotella Intermedia and Fusobacterium Nucleatum: An Ex Vivo Study. IOSR-JDMS.

[6] Mistry KS, Sanghvi Z, Parmar G, Shah S (2014). The antimicrobial activity of Azadirachta indica, Mimusops elengi, Tinospora cardifolia, Ocimum sanctum and 2% chlorhexidine gluconate on common endodontic pathogens: An in vitro study. Eur J Dent.

[7] Patil P, Patil S, Mane A, Verma S (2013). Antidiabetic activity of alcoholic extract of Neem (Azadirachta Indica) root bark. Natl J Physiol Pharm Pharmacol.

[8] Jerobin J, Makwana P, Suresh Kumar RS, Sundaramoorthy R, Mukherjee A, Chandrasekaran N (2015). Antibacterial activity of neem nanoemulsion and its toxicity assessment on human lymphocytes in vitro. Int J Nanomedicine.

[9] Botelho MA, dos Santos RA, Martins JG, Carvalho CO, Paz MC, Azenha C (2008). Efficacy of a mouthrinse based on leaves of the neem tree (Azadirachta indica) in the treatment of patients with chronic gingivitis: A double-blind, randomized, controlled trial. J Med Plant Res.

[10] Chatterjee A, Saluja M, Singh N, Kandwal A (2011). To evaluate the antigingivitis and antipalque effect of an Azadirachta indica (neem) mouthrinse on plaque induced gingivitis: A double-blind, randomized, controlled trial. J Indian Soc Periodontol.

[11] Dutta A, Kundabala M (2014). Comparative anti-microbial efficacy of Azadirachta indica irrigant with standard endodontic irrigants: A preliminary study. J Conserv Dent.

[12] Kaur K, Kumar T, Mittal S, Bansal R (2018). Phytomedicine: Herbal venture in green endodontics. Endodontology.

[13] Tasanarong T, Patntirapong S, Aupaphong V (2021). The inhibitory effect of a novel neem paste against cariogenic bacteria. J Clin Exp Dent.

[14] Chen H, Yuan J, Hao J, Wen Y, Lv Y, Chen L (2019). alpha-Humulene inhibits hepatocellular carcinoma cell proliferation and induces apoptosis through the inhibition of Akt signaling. Food Chem Toxicol.

[15] Pejin B, Kojic V, Bogdanovic G (2014). An insight into the cytotoxic activity of phytol at in vitro conditions. Nat Prod Res.

[16] Turkez H, Celik K, Togar B (2014). Effects of copaene, a tricyclic sesquiterpene, on human lymphocytes cells in vitro. Cytotechnology.

[17] Amiel E, Ofir R, Dudai N, Soloway E, Rabinsky T, Rachmilevitch S (2012). beta-Caryophyllene, a Compound Isolated from the Biblical Balm of Gilead (Commiphora gileadensis), Is a Selective Apoptosis Inducer for Tumor Cell Lines. Evid Based Complement Alternat Med.

[18] Hui LM, Zhao GD, Zhao JJ (2015). delta-Cadinene inhibits the growth of ovarian cancer cells via caspase-dependent apoptosis and cell cycle arrest. Int J Clin Exp Pathol.

[19] Afoulous S, Ferhout H, Raoelison EG, Valentin A, Moukarzel B, Couderc F (2011). gymnocephalum Essential Oil: Chemical Composition and Cytotoxic, Antimalarial and Antioxidant Activities, Attribution of the Activity Origin by Correlations. Molecules.

[20] Chahardehi AM, Arsad H, Ismail NZ, Lim V (2021). Low cytotoxicity, and antiproliferative activity on cancer cells, of the plant Senna alata (Fabaceae). Rev Biol Trop.

[21] Wang Z, Liu F, Yu JJ, Jin JZ (2018). beta-Bourbonene attenuates proliferation and induces apoptosis of prostate cancer cells. Oncol Lett.

[22] Yamaguchi M, Levy RM (2016). beta-Caryophyllene promotes osteoblastic mineralization, and suppresses osteoclastogenesis and adipogenesis in mouse bone marrow cultures in vitro. Exp Ther Med.

[23] Sharma C, Vas AJ, Goala P, Gheewala TM, Rizvi TA, Hussain A (2014). Ethanolic Neem (Azadirachta indica) Leaf Extract Prevents Growth of MCF-7 and HeLa Cells and Potentiates the Therapeutic Index of Cisplatin. J Oncol.

[24] Legault J, Pichette A (2007). Potentiating effect of beta-caryophyllene on anticancer activity of alpha-humulene, isocaryophyllene and paclitaxel. J Pharm Pharmacol.

[25] Thamizharasi G, Sindhu G, Veeravarmal V, Nalini N (2019). Preventive effect of D-carvone during DMBA induced mouse skin tumorigenesis by modulating xenobiotic metabolism and induction of apoptotic events. Biomed Pharmacother.

[26] Kushwaha P, Khedgikar V, Haldar S, Gautam J, Mulani FA, Thulasiram HV (2016). Azadirachta indica triterpenoids promote osteoblast differentiation and mineralization in vitro and in vivo. Bioorg Med Chem Lett.

[27] Kushwaha P, Ahmad N, Dhar YV, Verma A, Haldar S, Mulani FA (2019). Estrogen receptor activation in response to Azadirachtin A stimulates osteoblast differentiation and bone formation in mice. J Cell Physiol.

